# A Novel Approach for Sleep Arousal Disorder Detection Based on the Interaction of Physiological Signals and Metaheuristic Learning

**DOI:** 10.1155/2023/9379618

**Published:** 2023-01-13

**Authors:** Afsoon Badiei, Saeed Meshgini, Khosro Rezaee

**Affiliations:** ^1^Department of Biomedical Engineering, Faculty of Electrical and Computer Engineering University of Tabriz, Tabriz, Iran; ^2^Department of Biomedical Engineering, Meybod University, Meybod, Iran

## Abstract

The vast majority of sleep disturbances are caused by various types of sleep arousal. To diagnose sleep disorders and prevent health problems such as cardiovascular disease and cognitive impairment, sleep arousals must be accurately detected. Consequently, sleep specialists must spend considerable time and effort analyzing polysomnography (PSG) recordings to determine the level of arousal during sleep. The development of an automated sleep arousal detection system based on PSG would considerably benefit clinicians. We quantify the EEG-ECG by using Lyapunov exponents, fractals, and wavelet transforms to identify sleep stages and arousal disorders. In this paper, an efficient hybrid-learning method is introduced for the first time to detect and assess arousal incidents. Modified drone squadron optimization (mDSO) algorithm is used to optimize the support vector machine (SVM) with radial basis function (RBF) kernel. EEG-ECG signals are preprocessed samples from the SHHS sleep dataset and the PhysioBank challenge 2018. In comparison to other traditional methods for identifying sleep disorders, our physiological signals correlation innovation is much better than similar approaches. Based on the proposed model, the average error rate was less than 2%–7%, respectively, for two-class and four-class issues. Additionally, the proper classification of the five sleep stages is determined to be accurate 92.3% of the time. In clinical trials of sleep disorders, the hybrid-learning model technique based on EEG-ECG signal correlation features is effective in detecting arousals.

## 1. Introduction

An arousal during sleep is often thought of as a sleep disorder caused by fragmented sleep [1]. Changes in mobility, motor consciousness, and response readiness are caused by arousal-induced changes in sleep because they raise blood pressure and heart rate [2]. Multiple system atrophy (MSA), Lewy body disease (LBD), and Alzheimer's disease (AD) can all be detected early by studying physiological sleep signals [3]. The precise recognition of sleep stages and patterns can be crucial in reducing the progression of neurological diseases in such situations [4]. According to the American Academy of Sleep Medicine (AASM), monitoring physiological markers can detect rapid changes in sleep stages [5]. Consequently, classification of sleep stages, investigation of heart rate variability, REM sleep without atonia (RSWA), polysomnography (PSG), electrooculography (EOG), electroencephalography (EEG), electrocardiography (ECG), and electromyography (EMG) have received less attention in detecting arousal (EMG). To make easier the recognition of neurocognitive disorders, EEG is a cost-effective and promising test [6]. Through activation of the autonomic nervous system (ANS), cortical arousals are linked to fluctuations in heart rate and blood pressure [7–9]. Neurologists recognize arousals as abrupt changes in the EEG frequency [10], so they prefer to use EEG signal analysis to detect them. Although recording EEG data during sleep testing can be cumbersome for most individuals, most arousal episodes are associated with ANS activity. Sleep stage changes and other related problems are commonly accompanied by increased heart rate variability [11–13]. Arousals, also called cortical and autonomic activation, can occur spontaneously or as a result of respiratory dysfunction during sleep [14]. Physiological markers such as EEG and ECG and ANS activation during sleep arousal have been studied previously [15].

Nonlinear dynamical systems (NDSs) are thought to possess key properties based on their metric disparities between events [16]. Due to the complex patterns of such metric discrepancies, quantifying such differences is a difficult task for recognizing a correlation. Physiological sleep signals are assessed and managed nightly by neurologists [17], which is an empirical, inefficient, and time-consuming process [18]. Therefore, automatic interpretation and recognition are desirable.

Some previous research studies attempted to undertake automatic arousal detection based on physiological signals, with varying degrees of success. Attempts have been made to establish a link between physiological signals and sleep stage shifts [12, 13]. The study [14] has examined the relationship between arousal and the heart rate response to sleep stage changes. Despite the fact that the standard approach implies that at least one EEG channel is beneficial, some contributors have suggested that alternative physiological signals be used [19]. The difficulties of using a semiautomated system to detect and measure arousal have been highlighted in ECG signals [20]. Pillar et al. described an automated detection technique for separating AASM-defined arousals from sleep using peripheral arterial tonometry and a mobile device [21]. Warrick and Nabhan Homsi [22] proposed identifying sleep arousal using PSG and recurrent neural networks (RNNs). Deep learning (DL) has recently been widely employed to analyze EEG and ECG signals, including convolutional neural networks (CNNs) for sleep stage scoring [23–26], and an end-to-end (e2e) deep learning method based on multiphysiological inputs [27]. Tsinalis et al. [28] used time-frequency analysis and stacked sparse auto-encoders (AEs) to create an autonomous sleep stage grading system. Macias et al. [29] used the wavelet transform (WT) to classify physiological sleep data and then used neural network (NN) to detect alertness. Zhou et al. [30] reported a developed ensemble deep learning (EDL) structure that retains temporal associations of multimodal physiological inputs using a positional embedding-based multihead attention architecture. Ugur and Erdamar [31] used an ensemble bagged tree to assess quantitative sleep EEG synchronization for automatic arousals detection and achieved 99.5% accuracy. From PSG recordings, Karimi and Asl [32] proposed an automatic identification approach for nonapneic sleep arousal zones. They developed ensemble techniques and concentrated on feature subset selection and consensus approaches.

Nonlinear analysis of the EEG signal has long been recognized as providing information beyond that provided by regular EEG metrics [31]. Pathological events and normal cognition have both been shown to have different levels of brain complexity [32]. The chaotic phenomena are one of the primary causes of EEG signal complexity, and chaotic dynamics are characterized by sensitive dependency on initial conditions. Moreover, a Lyapunov exponent [33] is a method for describing the chaotic nature of EEG signals. In addition, some research has shown that the *R* segment of ECG signals can exhibit fractal behavior [34]. The classic and effective techniques for constructing discriminating features from physiological signals is single spectrum entropy analysis [35], entropy [36], complexity [37], Lyapunov exponent [38], fractal dimension [39], and wavelet transform [40].

Comparing existing studies in an unbiased and fair manner is very difficult because there are so many approaches to study the same subject [41, 42]. For datasets that are not publicly available, physiological signals and evaluation performance metrics differ. A major problem is the lack of standards for detecting sleep arousal, which is what creates this issue.

## 2. Motivation and Contribution

The frequent awakening from sleep, in addition to disrupted and fragmented sleep, is a precursor to somnipathy, which can lead to a variety of future health problems. Problems with sleep arousal have been linked to obesity, depression, heart disease, and diabetes. In order to improve public health, we need a deeper understanding of arousal neurophysiology.

It is possible to diagnose and treat sleep issues if excessive arousals are detected early. Early recognition of a patient's health condition can prevent changes in blood pressure and heart disease. Sleep arousal is often determined by PSG recordings. Furthermore, sleep-awakening events are subjectively assessed and vary greatly among professionals. In order to determine the level of arousal during sleep, sleep experts must examine PSG recordings. Clinicians would greatly benefit from an automated PSG-based sleep arousal detection system.

Two major obstacles must be overcome (1) understanding arousal disorders, which can cause disease, and the detrimental effects of excessive arousal on regular sleep. (2) The neurologist's outcome must be consistent if the model creates sleep stages and detects arousal disorders.

Taking care of these issues may have a substantial impact on the treatment process, and the individual may be cured of neurological ailments in the future. Thus, a precise approach for assessing arousals and sleep stages must be developed to monitor the diagnosis. Experts can significantly benefit from improving decision making through meta-learning and optimization methods in this context [43].

In this study, Lyapunov exponents, wavelet transforms, and fractals are used to quantify sleep and arousal stages through feature extraction from EEG-ECG signals. For the first time, an efficient hybrid-learning method is presented in this paper to detect and assess arousal incidents. In this novel model, a modified drone squadron optimization (mDSO) algorithm [44] is used to optimize support vector machines (SVMs) with radial basis functions (RBFs). As compared to other traditional methods for identifying sleep disorders, our physiological signals correlation innovation is much more effective. According to the proposed model, there was an average error rate of less than 2% for two-class issues and 7% for four-class issues, respectively.

A key contribution of this study is (a) the development of correlations between physiological signals, (b) the development of feature extraction methods, and (c) the design of the optimal classification scheme for the diagnosis of arousals, sleep stages, and sleep disorders.

**Figure 1:**
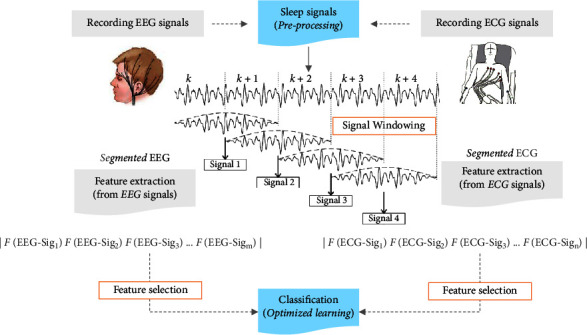
The framework of the proposed procedure.

We also present evidence supporting the utility of EEG and ECG signals with correlated brain and autonomic activity effects. The analyses of ECGs and EEGs enabled a more detailed understanding of arousal and sleep stages. An EEG-ECG system can also be used to quantify psychological sleep events such as arousal disorders and sleep stage changes as shown in Figure 1. It is common for physiological signals of arousal to change abruptly from deep to light sleep, which is why sleep stages were also identified. Due to this, the authors decided to classify sleep steps as well as determine arousal levels.

This paper is organized as follows: Section 3 introduces the learning structure.  Section 4 describes the method proposed for identifying abrupt changes and sleep stage. In Section 5, statistical analyses and results are discussed. Finally, conclusions are drawn in Section 6.

## 3. Learning Structure

To optimize the classification approach based on the learning process, this section discusses the components of the learning structure.

### 3.1. Classification Learning

Let us assume that we need to obtain a determination function *f* with the characteristic *f* (*x*_*j*_) = *y*_*j*_, ∀*j* [45].

(1) $$\begin{array}{c} y_{j}\left[\left(w.x_{j}\right)+b\right]\geq 1,\forall j\text{.} \end{array}$$

A discriminating hyper-plane typically does not exist. The slack variables *ζ*_*j*_ are offered to provide for the event of examples violating (1):

(2) $$\begin{array}{c} \zeta \geq \;\;0\forall j\text{.} \end{array}$$

Moreover, **w** is a weight vector, **x** is the input vector, and **b** is bias. As a result, we have

(3) $$\begin{array}{c} yj\left[\left(w.xj\right)+b\right]\geq 1-\zeta j,\forall j\text{.} \end{array}$$

The support vector (SV) procedure to minimize the assured uncertainty bound includes

(4) $$\begin{array}{c} \tau \left(w,\zeta \right)=0.5\times \left(w\;.w\right)+\gamma \overset{l}{\underset{j=1}{\sum }}\zeta_{j}\text{.} \end{array}$$

The soft margin equation is made by multiplying *ζ* by a hyperparameter *γ* and adding two terms with *l* training points. The minimization procedure is conducted based on mentioned constraints in (2)–(3). Importing Lagrange multipliers *α*_*j*_ and applying the Kuhn–Tucker theory of the optimization approach, the answer can be determined

(5) $$\begin{array}{c} w=\sum_{j=1}^{l}y_{i}\alpha_{j}x_{j}\text{.} \end{array}$$

This condition in (3) must be precisely met by the relevant cases (*x*_*j*_*and y*_*j*_) in order for *α*_*j*_ to be nonzero. These **x**_**j**_ are known as “support vectors” and the rest of the training examples are unnecessary. Their exclusion from the expansion (5) is due to the fact that (3) is immediately satisfied (with *ζ*_*j*_ = 0). Calculating *α*_*j*_'s coefficients entails solving the quadratic programming issue as shown. Thus, the maximization of the following equation can be applied with regard to (7):

(6) $$\begin{array}{c} w\left(\alpha \right)=\sum_{j=1}^{l}\alpha_{j}-\frac{1}{2}\overset{l}{\underset{j,k=1}{\sum }}\alpha_{j}\alpha_{\;k}y_{j}y_{k}\left(x_{j}.x_{k}\right)\text{,} \end{array}$$

(7) $$\begin{array}{c} 0\leq \alpha_{j}\leq \gamma ,i=1,\ldots ,l,\overset{l}{\underset{j=1}{\sum }}\alpha_{j}y_{j}=0\text{.} \end{array}$$

Based on the dot product's linearity, the decision function can also be constructed as follows:

(8) $$\begin{array}{c} f\left(x\right)=sgn\left[\overset{l}{\underset{j=1}{\sum }}y_{j}\alpha_{j}\left(x.x_{j}\right)\right]+b\text{,} \end{array}$$

where, the sgn corresponds to the sign function or signum function. A set of input vectors *x*_1_,…, *x*_l_ can be nonlinearly translated into a high-dimensional feature space to provide significantly more general decision surfaces. The decision-making function is

(9) $$\begin{array}{c} f\left(x\right)=sgn\left[\overset{1}{\underset{j,k=1}{\sum }}y_{j}\alpha_{j}.K\left(x.x_{j}\right)\right]+b\text{.} \end{array}$$

As a result, kernel RBF can be displayed as the following for support vectors:

(10) $$\begin{array}{c} K\left(x\;,x_{j}\right)=\exp\;\left(-\left\|x-x_{j}\right\|\right)\times {\left(2\sigma^{2}\right)}^{-1}\text{.} \end{array}$$

### 3.2. Drone Squadron Optimization

The drone squadron optimization (DSO) is comprised of two components: semiautomated drones that conduct environmental surveys and a command center that processes data and updates the drones' firmware system as needed. The self-adaptive component of DSO used in this work is the perturbation/motion method used to get the target coordinates [44]. The command center devised this method of customizing the DSO to the search perspective during the global optimization phase.

Drones' capacity to be changed or modified in both hardware and software is one of its most distinguishing features (the firmware). As a result, researchers may quickly include new processes into the algorithm because these devices are controlled by software (firmware). The command center serves as a single point of contact for issuing orders and overseeing assigned responsibilities. Internal decisions are made based on the system's processing of inputs and data. The command center can update the drone software at any time, adapting a team's behavior to the scenario dynamically.

Drone squadrons and command centers make up the DSO algorithm. The command center accomplishes two goals by utilizing data collected by/from drones. To keep some control over the search process while also developing new drone-flying technology. Drones are not the answer because they are programmed to fly to a specific location (the actual solution). It consists of the methods and parameters used by teams when doing landscape searches.

There must be a *departure* point in order to avoid limiting the search area towards the origin assuming that the *offset* value is zero, the *departure* coordinates still have noise app lied to them, which causes a neighborhood search. If a firmware update condition is reached (for example, the number of iterations), the command center substitutes variations of the *w* best firmware for the *w* worst firmware. For *w*=1, this indicates that the team with the lowest cumulative rank will obtain a firmware version (random subtree replacement) of the best team.

## 4. Proposed Methodology

**Figure 2:**
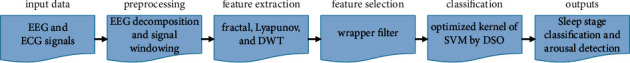
The schematic of the proposed method for detecting the arousal events and sleep stages.

Feature extraction, feature selection, and optimized classification are steps of the proposed strategy (Figure 2).

### 4.1. Preprocessing

We must window nonstationary physiological signals in order to conduct the essential analysis. Each window overlaps the previous one to preserve the information's nature. Due to the fact that EEG and ECG signals vary in length across states, we separated them into equal segments based on the sampling frequency. In other words, we used the windowing technique, which divides the signal into equal time segments. By considering a 20%–50% overlap between two consecutive windows, the length of each window must be calculated in such a way that appropriate features may be extracted based on the established step-times [46]. The rationale for selecting the signal length and the percentage of overlap between the consecutive signals will be discussed in detail in the section on experimental results.

By generating deep sleep in the third stage and allowing it to take its course naturally in the fifth stage, band separation is accomplished utilizing EEG signals. The third stage is defined by the presence of slow, deep brain waves termed waves. We created low-pass filters with passing frequencies of 3 Hz or fewer to differentiate EEG signals from waves. Individuals' attentiveness decreases during the deep sleep stage, which means that nearby noises or activities may go unnoticed. This stage serves as a transition between awake and deep sleep. Dreaming typically happens at step 5, sometimes referred to as rapid eye movement (REM). Deep sleep, which is frequently referred to as partial sleep, is defined by increased brain activity, eye movement, and rapid breathing. Using the linear regression algorithm (LRA), we can similarly distinguish cardiac complexes from signals [47].

### 4.2. Feature Extraction and Selection

The feature extraction level includes three descriptors as follows:

#### 4.2.1. Lyapunov's Exponent

In phase space, we can determine the convergence or average exponential divergence of surrounding trajectories by using Lyapunov exponents [48]. Generally, chaotic systems have a positive Lyapunov exponent. Consider $\dot{p}=f\left(p\right)$ indicates the *N*-dimensional state vector *p* of a dynamic system. As the system progresses, we measure the evolution of the distance *d* between the adjacent phase points *p*_1_ and *p*_2_ in the dimensional space and generate trajectories [*p*_1_ (*t*), *p*_2_ (*t*)]:

(11) $$\begin{array}{c} d\left(t\right)=\left|p_{1}\left(t\right)–\;p_{2}\left(t\right)\right|\text{,}\\ =\left|\overset{⟶}{\varepsilon }\left(t\right)\right|\text{,} \end{array}$$

where $\left|\overset{⟶}{\;\varepsilon }\left(t\right)\right|$ is a very small and positive value. When chaotic dynamics are present in system $\dot{p}=f\left(p\right)$, *d* (*t*) will increase exponentially

(12) $$\begin{array}{c} d\left(t\right)\approx d\left(0\right)e^{\lambda t.} \end{array}$$

Using the average velocity of the trajectories as a starting point, we can determine the exponential divergence of the average velocity:

(13) $$\begin{array}{c} \lambda \approx \frac{\left[\ln\left(d\left(t\right)/d\left(0\right)\right)\right]}{t}\text{.} \end{array}$$

In exact terms

(14) $$\begin{array}{c} \lambda =\frac{\underset{\begin{array}{c} t⟶\infty \\ d\left(0\right)⟶0 \end{array}}{\lim}\left[ln\left(d\left(t\right)/d\left(0\right)\right)\right]}{\;t}\text{.} \end{array}$$

The following matrix shows the extracted features from overlapped-windowed EEG/ECG signals. We form two independent feature vector matrixes for each physiological signal. Following is an example using the Lyapunov exponent and a sample feature extraction vector from each windowed signal based on three descriptors.

(15) $$\begin{array}{c} \frac{EEG}{ECG\;Feature\;\;Vector}=\left|\begin{array}{ccccc} F\left({Sig}_{1,1}\right) & F\left({Sig}_{1,2}\right) & F\left({Sig}_{1,3}\right) & \ldots & F\left({Sig}_{1,n}\right)\\ F\left({Sig}_{2,1}\right) & F\left({Sig}_{2,1}\right) & F\left({Sig}_{2,3}\right) & \ldots & F\left({Sig}_{2,n}\right)\\ \ldots & \ldots & \ldots & \ldots & \ldots \\ \ldots & \ldots & \ldots & \ldots & \ldots \\ F\left({Sig}_{m,1}\right) & F\left({Sig}_{m,2}\right) & F\left({Sig}_{m,3}\right) & \ldots & F\left({Sig}_{m,n}\right) \end{array}\right|\text{.}\\ An\;example\;based\;on\;the\;time\;series\;in\;Lyapunov^{\prime }s\;exponent=\left|\begin{array}{cccccc} d & & & & & \\ 2 & 0.42962 & -1.61402 & & & \\ 3 & 0.41118 & -088236 & -1.61117 & & \\ 4 & 0.40998 & 0.49658 & -1.59837 & -1.87389 & \\ 5 & 0.83478 & 0.42890 & -0.88561 & -1.58723 & -1.98871 \end{array}\right|. \end{array}$$

#### 4.2.2. Fractal

The development of fractals occurs with repetitions, meaning that they are regularly deformed and reliant on the starting position. There are many solutions for implementing stochastic fractal dimensions, particularly Higuchi and Katz's fractal dimensions. These features are expressed in the fractal space as follows:

*(A) Correlation Dimension*. In the fractal theory of a signal, the correlation dimension (denoted by *v*) is a measure of the dimensions of space occupied by a set of random points, which is known as the fractal dimension. In the first dimension, the distance between every two-point pairs in feature space is obtained based on the phase environment. In the second step, the summation of *C* (*N* and *r*) and *D*_2_ parameters are estimated according to

(16) $$\begin{array}{c} {}_{C\;}{\left({\left(\right)}_{N\;,r}\right)}_{=}\frac{{}_{1}}{{}_{N\;}\left({\left(\right)}_{N-1}\right)}\overset{N}{\underset{i=1}{\sum }}\overset{N}{\underset{j=1}{\sum }}\theta \left(r-\underset{j\neq i}{\left\|v\left(j\right)-v\left(i\right)\right\|}\right)\text{,} \end{array}$$

where

(17) $$\begin{array}{c} \theta \left(x\right)=\left\{\begin{array}{cc} 0\text{,} & if\;x<0\text{,}\\ 1\text{,} & if\;x\geq 0\text{,} \end{array}\right. \end{array}$$

(18) $$\begin{array}{c} {D\;}_{2}=\lim_{r⟶0}\frac{\log_{2}\;C\left(N,r\right)}{\log_{2}\left(r\right)}\text{,} \end{array}$$

where ||.|| represents the Euclidean distance and *r* is the radius of the *V* (*j*) neighborhood. Also, *N* represents the number of points*, θ* is a step function, and finally, *V* is the phase-space vector, which is determined according to the following

(19) $$\begin{array}{c} V_{m}\left(i\right)=x\left(i\right),x\left(i+\tau \right),\ldots ,x\left(i+\left(m-1\right)\tau \right)\text{.} \end{array}$$

In this equation, *m* is the embedding dimension, and *t* is the time delay.

*(B) Fractal Dimension*. The degree of complexity, irregularity, and chaos of the signal is determined by the fractal dimension.

#### 4.2.3. Wavelet Descriptor

The discrete wavelet transform (DWT) was created because of the need for multiscale feature representation. DWT reflects physiological information in a variety of ways, depending on the size or scale. Selecting decomposition levels based on the frequency component that dominates the EEG and ECG data. A high degree of correlation between the levels of the signal sections and the frequency data are essential for categorizing signals. For our experiment, the study uses a five-level decomposition structure. As a result, physiological signals are divided into *D*1–*D*5 (detail coefficients) specialties and an *A*5 (approximation coefficients) approach. Wavelet types are typically investigated until the most appropriate one is found. A Daubechies wavelet is more suited for detecting signal changes because of its second-order (db2) smoothing characteristics. Therefore, db2 was used to compute the wavelet coefficients for this study.

#### 4.2.4. Feature Selection

Selecting features based on generic filters, we can use the shared wrapper feature selection as a recursive feature eradication for clusters, including the *k*-nearest appropriate neighbor and support vector machine. To do so, we allocate a score called *Sc*_*i*_ to each group, as explained by the following. Groups are then ranked relative to these assigned scores, and top-ranking groups are identified as the primary outputs.

(20) $$\begin{array}{c} {Sc}_{i}=\left(\delta_{i+}-\epsilon_{i+}\right)\times {\left(\delta_{i-}-\epsilon_{i-}\right)}^{-1}\text{,} \end{array}$$

where *δ*_*i+*_ and *ε*_*i+*_ are the mean and standard deviation of features associated with abrupt changes in the sleep stage, and *δ*_*i*−_ and *ε*_*i*−_ are the mean and standard deviation normal change patterns in the sleep stage, respectively.

### 4.3. Optimized Learning

A hybrid classification model based on the SVM learning repository is used in the first part. The initialization of the *γ* (i.e., is the inverse of the standard deviation parameter in the kernel) and *C* (a soft margin parameter) in the classification kernel is randomly generated for each classifier. The speed of change of each of these qualities is different. The modified DSO method is formed with a selected structure based on the least amount of error in the training data. This method accelerates and fine-tunes convergence toward the optimum response. This technique is repeated five times to evaluate the classification fusion of the pool structure, the average accuracy, and the best matching parameters. The network accuracy is a key factor in determining an appropriate match. Calculating the optimal values of the RBF kernel helps to search for the modified DSO algorithm for global optimizations.

Scaling, remodeling, and connection methods are used in the traditional DSO strategy, as well as selecting lower-quality solutions when stagnation occurs and firmware updates. The initial unpredictability of the procedure, as well as the occurrence of desperate portions in the search region, are, however, flaws. To reduce unpredictability and avoid desperate sections of the search region, we employ *k*-nearest neighbor (*k*-NN) clustering technique. The DSO algorithm's initialization challenge is solved, enabling the filtering of incorrect initialization and, as a result, an improvement in the final response. The K-NN classifier significantly aided in the determination of optimal starting values while using the standard DSO technique. *K* can be unlimited in the *k*-NN algorithm, which indicates that all points in the data are used to make the prediction, although their effect is inversely proportional to their Euclidean distances from the initial optimal targets. The following equation illustrates the predictor, where is the index of the *k*-NN

(21) $$\begin{array}{c} Error\left({wig}_{p}\right)=\underset{\begin{array}{c} q\in D_{K}\\ q\neq p \end{array}}{\sum }\frac{1}{{di\;st}_{Eucli}\left({wig}_{p},{wig}_{q}\right)+\varepsilon }Error\left({wig}_{p}\right)\text{.} \end{array}$$

The traditional DSO technique was repeated numerous times with varying values to create improper and appropriate starting points for use in the *k*-NN learning process, which were labeled 1 and 2, respectively, in proportion to the ultimate accuracy gained by establishing the best RBF kernel parameters.

### 4.4. Physiological Signal Correlation

Extracted features from five frequency bands are used to explore the correlation between EEG and ECG data for sleep stages and arousal detection. A significant relation is indicated by a positive correlation of selected parameters, whereas a negative correlation implies an inverse relationship. The EEG signal's estimated capabilities include five frequency bands delta (4.5–5.0 Hz), theta (5.5–8.5 Hz), alpha (5–5.9 Hz), sigma (13–16.5 H), and beta (13–16.5 H) (17–30 Hz). The spectral power of ECG signals was investigated at five different frequencies ranging from 0.33 Hz to 0.4 Hz. Normalizing signals at three levels of low (LF), high (HF), and very low (VLF) frequencies was also used to synchronize them. Different signals acquired from each sleep cycle were analyzed for correlation, which naturally necessitates detecting and evaluating sleep stages.

## 5. Experimental Results

### 5.1. Dataset

The proposed technique was tested using the Sleep Heart Health Study (SHHS) and PhysioBank databases, which were part of the Challenge 2018 project. The SHHS consists of arousal events and various transitions from deep sleep to light sleep. The National Heart, Lung, and Blood Institute has launched the SHHS, a multipurpose cohort study, to determine cardiovascular and other respiratory outcomes in the absence of any sleep disturbance. Between November 1, 1995, and January 31, 1998, 6,441 men and women over the age of 40 were registered for the SHHS visit.

**Figure 3:**
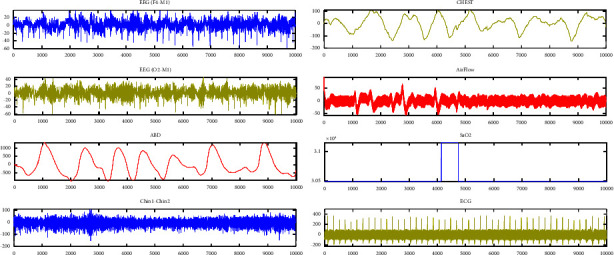
Various physiological signals of the human body to detect arousal during sleep are displayed. The most important signals that provide the highest classification accuracy for discrimination are the EEG and ECG signals.

As shown in Figure 3, physiological signals such as ECG, EEG, ABD, SaO2, chest, and AIRFLOW were annotated by neurological experts from numerous participants in both data and distinct periods. Wake state (*W*), sleep stage 1 (*S*1), sleep stage 2 (*S*2), sleep stage 3 (*S*3), sleep stage 4 (*S*4), and REM sleep were all given abbreviations. In both databases, the events captured in the crucial EEG signal are in the form of arousal. The cardiac signal's duration is measured in minutes and seconds, just like the EEG signal. During the incident, the percentage decrease in the amount of dissolved oxygen in the blood was also recorded. The lowest levels of dissolved oxygen in the blood were seen in general. Each record comprised all of the comments from the specialist's manual registration files that could be identified based on the type of occurrence or sleep stage.

### 5.2. Setting

To run the algorithms in Matlab 2019b simulation environment, a computer system with 8 GB of RAM and a 64-bit operating system was required for Intel (R), Core (TM), and Core i7 CPU. The synchronization of both signals acquired from the volunteers' sleep phases, which included six sleep steps, was considered equal to identify the sleep stages due to the significant duration of specific signals obtained from the participants.

The arousal events classification problem is a two-class task for EEG, ECG, and EEG-ECG signals. All ECG and EEG signals were preprocessed into discrete windows (at 1-second intervals) and deep sleep bands, with levels in the ECG signal that were higher than the EEG signal, however proportionate to it. It was extracted at the feature extraction stage along with Katz and Higuchi features (extraction from all separated signal frames) the following features from EEG and ECG signals: correlation, entropy, energy, skewness, standard deviation, mean, power ratio, spectral power, zero crossing, and reverse of differentiation (with vector features ranging in length from 47 to 60).

### 5.3. Assessments

In the assessment step, we partition data into test and training data using K-fold cross-validation (CV). In all, the signals were divided into four groups. The semiconscious and awakening periods (also known as the EEG signal with beta and alpha sub-bands) as well as their homologous time intervals in the ECG signal set were deleted from brain datasets. The first signifies a rapid transition from light to deep sleep (Class 1). The second class denotes deep sleep (Class 2), the third class denotes low-intensity sudden deep-sleep type shifts (Class 3), and the fourth class denotes rapid deep-sleep style changes (Class 4). In the first report of outcomes, we compare learning based on particle swarm optimization (PSO), genetic algorithm (GA), and DSO algorithms with mDSO algorithm in improving the SVM classifier. As shown in Tables 1 and 2, we compared the performance of the SVM classifier in classes two and four using the mDSO optimizer to alternative optimization strategies. As demonstrated in Table 1, the binary classification indicated whether or not there was a sudden transition from deep to light sleep.

**Figure 4:**
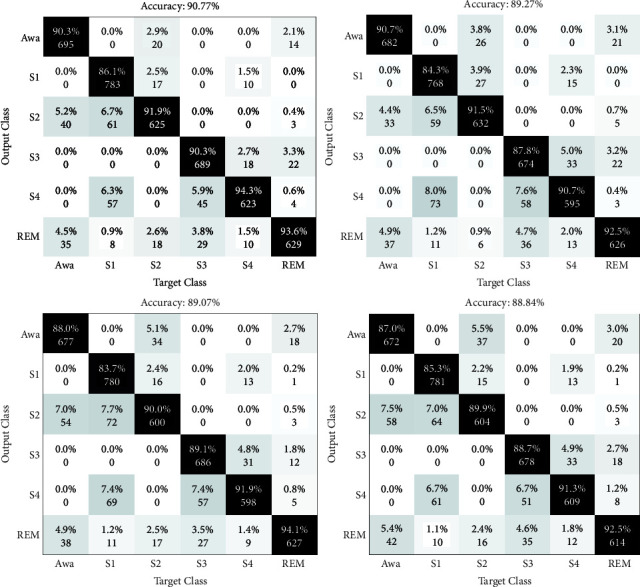
The confusion matrix is depicted in this table, which includes the first (best) and second (worst) rows of the two-fold from the 10-folds of EEG and ECG signals in the first dataset.

**Figure 5:**
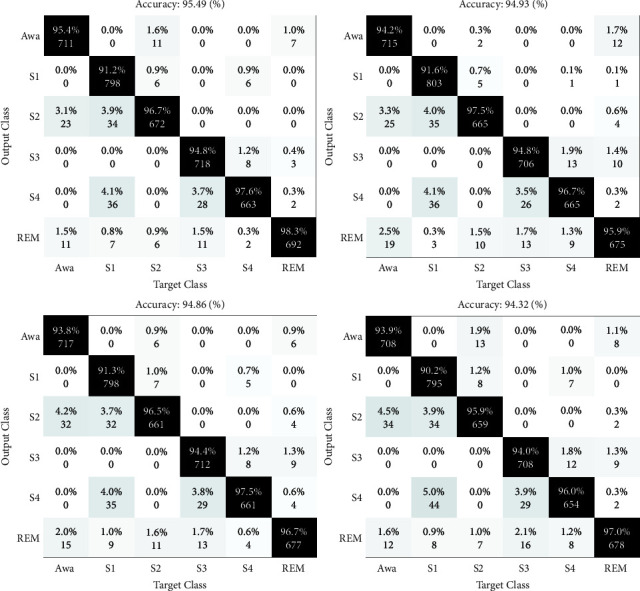
The confusion matrix is depicted in this table, which includes the first (best) and second (worst) rows of the two-fold from the 10-folds of EEG and ECG signals in the second dataset.

**Figure 6:**
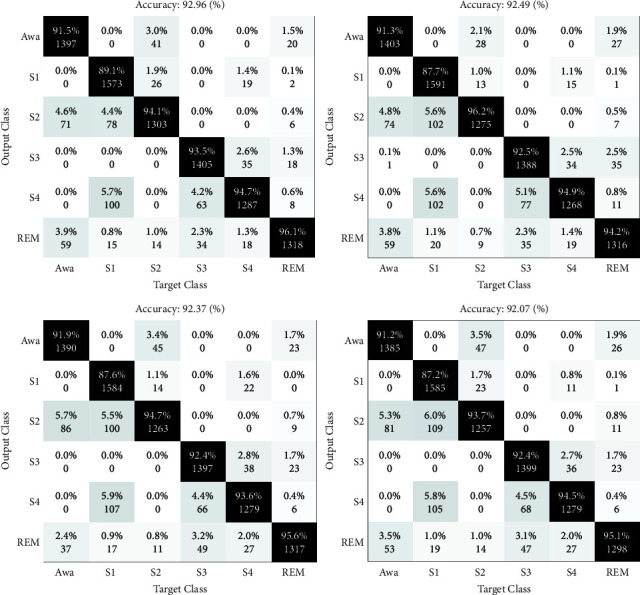
The suggested method's confusion matrices in the concatenating state of the derived features from both physiological signals in both datasets.

The results of the sleep stages classification are depicted as confusion matrices in Figures 4–6. Four folds are available in these figures, with two maximum and two minimum results, and the CV is preferred at 10.

The matrices shown in Figures 4 and 5 are the results of the sleep stage classification on datasets 1 and 2. The classification outcomes of sleep stages are displayed as confusion matrices in Figures 4–6. The outputs of applying the approach to both datasets are also shown in Figure 6.

**Figure 7:**
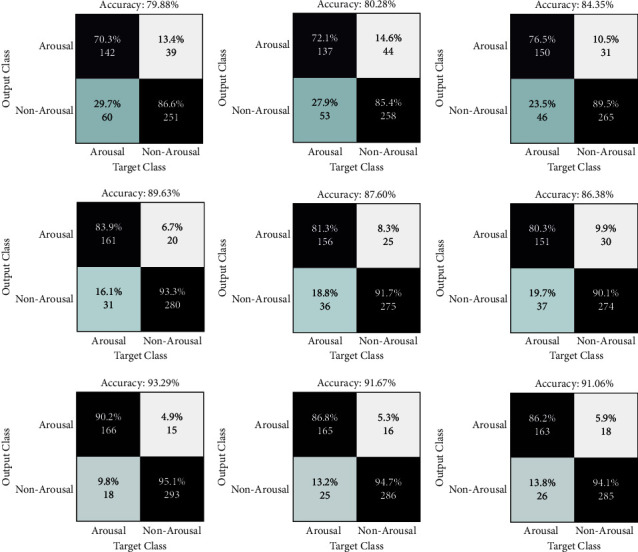
For ECG (first raw), EEG (second raw), and EEG-ECG (third raw) signals, the results of a confusion matric (best three-folds) for the classification of arousal and nonarousal events are shown.

HRV analysis has been employed for decades to assess changes in the autonomic nervous system (ANS), which is the balance between the parasympathetic and sympathetic nervous systems. A change in the heart rate is one of the main indicators of cerebral cortex arousal [49–55]. Experts have identified a stronger correlation between clinical outcomes and heart rate analysis, which sheds light on the systemic effects of arousal and sleep phases. The findings in this figure represent the results of using the suggested method to both datasets, which were evaluated by concatenating the extracted features from both signals. Only the experiment's results are shown in these figures. To prevent overfitting in learning, the data are also shuffled. The suggested method's sleep stage classification findings are included in the confusion matrix. The primary purpose of presenting repeats is to demonstrate the degree of the apparent variance among the acquired data by computing the standard deviation (STD), which is used to verify the method's generalizability and robustness in diagnosing arousal. The first dataset's distribution accuracy is higher, while the divergence in the second dataset's results is minor. The results of the classification of arousal and nonarousal events for EEG, ECG, and EEG-ECG signals are shown in Figure 7. Arousal may be identified with a greater precision in the presence of both signals and due to their correlation, i.e., the concatenation of information from both signals.

The comparison is based on the algorithm being applied to both datasets. The appearance of arousal events is also scored by a neurologist. The accuracy, AUC, and recognition rate (RR) were assessed, and the cross-validation approach was investigated by setting CV = 10. Tables 3–5 illustrate the accuracy, AUC, and RR of feature selections obtained from FS, DWT, and LE descriptors of arousal events under various feature selection settings.

As shown in Table 5, all processes of feature extraction, feature selection, and classification were analyzed employing an improved SVM under various situations. These tables show how to use classification and apply features to EEG or ECG signals in a variety of situations.

## 6. Discussion

**Figure 8:**
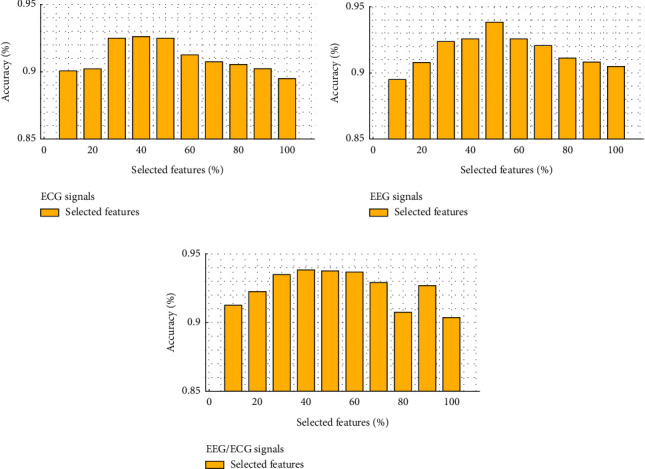
Three tests were conducted to determine which aspects of (a) ECG, (b) EEG, and (c) EEG/ECG signals were most efficient in detecting arousal disorder.

Three experiments are provided in Figure 8 to assess the influence of concatenated features of (a) ECG, (b) EEG, and (c) EEG-ECG signals. In the distance of 30–50% of all features, the features are selected because a proper discriminated effect in the classification.

**Figure 9:**
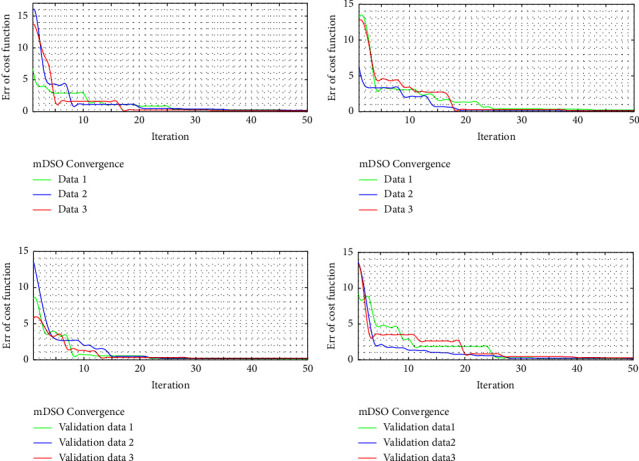
The comparison of mDSO convergence based on the cost function error calculation. The mDSO is a speedy algorithm that can quickly reach the ideal value. As a result, we employ SVM for reaching optimal classification conditions by adjusting data. (a, b) Test data and (c, d) validation data applied to the improved classifier.

For both the EEG-ECG signals, the FS, DWT, and LE descriptors retrieved a total of 112 features, of which 34–40 concatenated features were chosen as the best subset features in Figure 8(c). The effective features, depending on the signals, have a higher impact on the final classification accuracy and are critical to the arousal diagnosis in all three trials shown in Figure 8. For identifying sleep disorders, the same is true, with 30–50 percent of the selected criteria yielding the highest classification accuracy. Different types of data, such as test and validation signals, were analyzed. As illustrated in Figure 9, the convergent level of the error is calculated using the loss function over a finite number of epochs. The mDSO procedures illustrated convergence of optimizing the SVM classifier is rapid and reliable for both test and validation data, as seen in this image. As a result, we investigate SVM in order to achieve optimal classification conditions by altering data.

**Figure 10:**
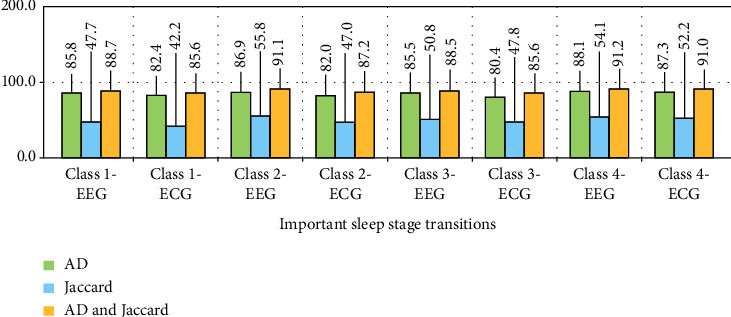
Based on AD, Jaccard, and AD-Jaccard calculations for ECG and EEG signals, the effect of characteristics in crucial stages of the sleep transition (from class 1 to class 4, respectively) was evaluated.

The Jaccard and average degree (AD) benchmarks are shown in Figure 10 to measure the quality of extracted features from all three FS, DWT, and LE descriptors, which are used to test the feature integration.

**Figure 11:**
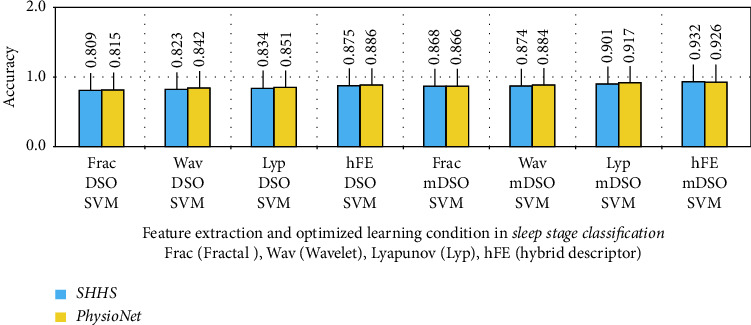
The results are compared as a bar-plot based on combined features for the sleep stage classification.

**Figure 12:**
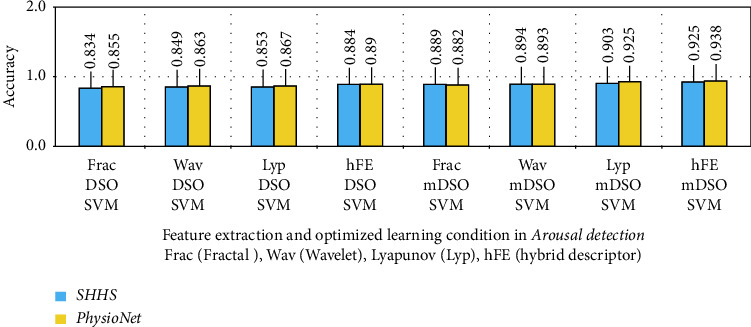
The results are compared as a bar-plot based on combined features for arousal detection.

The findings of its two-stage repeat, as well as for both EEG and ECG physiological signal models, show that it has a good performance in identifying sleep stage alterations. The most effective classes for identifying transitions in sleep stages (Class 1 to Class 4) for EEG-ECG data are presented in this comparison (Class 1 to Class 4). What was thought to be a 5-repeat accuracy criteria experiment in arousal disorder detection turned out to be inconsequential, with modest variations recorded. The outputs from the usage of the hybrid descriptor and mDSO procedure between EEG/ECG signals were more significant with the neurologistss' opinions, as shown in Figures 11 and 12. The results are compared as a bar-plot based on combined features in Figure 11 for the sleep stage classification.

Two different types of data are used to assess the method's performance: EEG and ECG signals. The advantages of the mDSO method over other methods for increasing learning include its confidence in achieving the optimal response and its rapid convergence.

Unlike prior studies that concentrated exclusively on arousal, the current research separated the significant stages of sleep to assess the intensity and intervals of arousal events. Other methods have either used cardiac signals or evaluated only EEG signals, with no consideration of the factors underlying their association in the response. Accuracy levels higher than 90% for different classes of sleep stages, and thus identifying arousal events using traditional algorithms, require the extraction of precise patterns of features.

Some algorithms in this field have been successful in classifying arousal episodes; nevertheless, issues such as generalizability and computational complexity for big data still exist [25, 26, 56–60]. There has not been a study of the simultaneous recognition of sleep stages, arousal detection, and the correlation between EEG and ECG signals.  Table 6 analyses similar arousal detection approaches and compares the performance of the suggested method-based measures such as accuracy.

We used multiple signal channels and employed the method on a large number of similar datasets, whereas analyzing features and connecting signals using single-channel signals is a challenge that must be addressed to ensure precision, both in the detection of sleep stages and in arousal events of varying intensities [23, 31]. To extract useful features, we employed a hybrid approach. Deep learning approaches [23–26, 61] may be able to extract automated separable features for the categorization. Nonetheless, extensive data and prolonged training complicate the signal analysis, i.e., the identification of hyperparameters and variables of training, high susceptibility to the uncertainty effect, and similar items in variation analysis parameters. What is referred to as arousal detection in EEG and even ECG analyzing may yield interpretations other than arousal based on signals such as PSG [22, 32, 60]. As a result, the proposed technique has a significant advantage over similar methods that uses single nonstationary and noise PSG or EEG signals [25, 26, 59] to detect sleep stages. Thus, by identifying combined features deduced from signals with richer EEG and ECG information for accurate arousal analysis.

Study limitations included a limited number of patients, complex physiological signals, and inaccurate labeling when it came to identifying arousal. Moreover, there is a lack of confirmation of a relationship between physiological signals during arousals, lack of an indicator boundary between arousals, and insufficient substrate to the estimate arousal intensity. Noise, including motion noises, is one of the biggest challenges in arousal and sleep stages detection. Since the EEG signal also includes artifacts such as respiration signals and movement signals, detecting the signal's noise component can be challenging. A second type of noise is produced when the power frequency of the recording equipment coincides with that of the well-known noise [62]. Even though we extracted features using several methods and windowed the signal, it is proposed that the method, in addition to filtering the signal based on previous suggestions regarding the signal decomposition using efficient methods such as wavelet [63], also prevents information from being lost by physiological signals by preventing information from being lost. In the future, authors will use graph convolutional network (GCN) [64] and improved transfer learning networks [65] to determine arousal events from EEG to ECG data.

## 7. Conclusion

This paper provided an effective approach for extracting relevant characteristics from EEG to ECG data and categorizing them using optimal learning. In addition, we develop a metaheuristic learning model based on physiological sleep signals to detect arousal disorders. This study also intends to define distinct sleep stages as well as rapid transitions from deep to light sleep stages, which is a short-term abnormality in EEG-ECG signals. Nonetheless, it offers critical information on the likelihood of developing Alzheimer's, Parkinson's, or MSA illnesses. There is strong evidence of a relationship between these problems and the diseases indicated above. The arousal events were described using an optimal learning model based on the mDSO and SVM. Using concatenated features, we were able to develop an efficient discriminative approach that analyzed physiological signals using three descriptors. Future research could look into the relationship between ECG and EEG signals as a way to prevent numerous diseases caused by sleep disturbances.

## Figures and Tables

**Table 1:** The error assessment of sleep steps classification for a two-class problem using EEG signals.

Repetitions	SVM		SVM (RBF)		SVM (RBF) + GA		SVM (RBF) + PSO		SVM (RBF) + DSO		SVM (RBF) + mDSO	
	Min	Max	Min	Max	Min	Max	Min	Max	Min	Max	Min	Max
10-fold (1)	0.11	0.15	0.08	0.11	0.08	0.12	0.05	0.08	0.03	0.05	0.02	0.04
10-fold (2)	0.09	0.17	0.08	0.12	0.09	0.12	0.06	0.09	0.04	0.08	0.02	0.05
10-fold (3)	0.15	0.18	0.09	0.13	0.08	0.12	0.06	0.08	0.04	0.06	0.01	0.05
10-fold (4)	0.11	0.16	0.08	0.12	0.06	0.13	0.06	0.08	0.02	0.07	0.03	0.05
10-fold (5)	0.14	0.17	0.11	0.13	0.11	0.12	0.08	0.08	0.04	0.08	0.02	0.04
10-fold (6)	0.13	0.18	0.10	0.14	0.10	0.11	0.09	0.09	0.04	0.06	0.01	0.03
10-fold (7)	0.11	0.16	0.10	0.14	0.09	0.11	0.06	0.11	0.03	0.06	0.01	0.03
10-fold (8)	0.09	0.16	0.07	0.14	0.07	0.09	0.05	0.08	0.05	0.07	0.02	0.03
10-fold (9)	0.10	0.14	0.12	0.13	0.11	0.11	0.09	0.07	0.05	0.09	0.03	0.05
10-fold (10)	0.12	0.15	0.10	0.13	0.09	0.09	0.07	0.12	0.04	0.08	0.02	0.04
Av	0.115	0.162	0.093	0.129	0.088	0.112	0.067	0.088	0.038	0.071	**0.019**	**0.041**

Bold values are the best values obtained.

**Table 2:** The error assessment of sleep steps classification for a multiclass problem using EEG signals.

Repetitions	SVM		SVM (RBF)		SVM (RBF) + GA		SVM (RBF) + PSO		SVM (RBF) + DSO		SVM (RBF) + mDSO	
	Min	Max	Min	Max	Min	Max	Min	Max	Min	Max	Min	Max
10-fold (1)	0.24	0.31	0.19	0.28	0.16	0.24	0.10	0.22	0.12	0.16	0.08	0.12
10-fold (2)	0.24	0.37	0.19	0.30	0.14	0.26	0.14	0.23	0.13	0.18	0.06	0.14
10-fold (3)	0.21	0.28	0.24	0.32	0.19	0.21	0.16	0.24	0.14	0.17	0.05	0.11
10-fold (4)	0.27	0.30	0.24	0.28	0.20	0.22	0.16	0.21	0.15	0.19	0.06	0.12
10-fold (5)	0.23	0.33	0.18	0.24	0.16	0.23	0.18	0.21	0.14	0.20	0.08	0.13
10-fold (6)	0.24	0.34	0.22	0.32	0.18	0.25	0.16	0.21	0.15	0.19	0.12	0.14
10-fold (7)	0.25	0.27	0.22	0.28	0.16	0.19	0.14	0.17	0.14	0.19	0.10	0.12
10-fold (8)	0.25	0.30	0.19	0.28	0.16	0.20	0.16	0.19	0.15	0.20	0.09	0.12
10-fold (9)	0.22	0.25	0.17	0.24	0.14	0.17	0.15	0.19	0.14	0.19	0.09	0.13
10-fold (10)	0.27	0.33	0.24	0.29	0.19	0.23	0.14	0.21	0.12	0.18	0.06	0.09
Av	0.242	0.308	0.208	0.283	0.168	0.220	0.133	0.208	0.138	0.185	**0.079**	**0.122**

Bold values are the best values obtained.

**Table 3:** The accuracy, AUC, and RR of the subset feature selection from the fractal descriptor are calculated under various scenarios. The results of SVM and improved SVM classifiers are compared to detect arousal events.

Signal type	No. of features	SVM			Optimized SVM		
		ACC (%)	AUC (%)	RR (%)	ACC (%)	AUC (%)	RR (%)
EEG	16	74.88	75.64	73.11	83.31	84.19	83.21
	24	79.54	77.32	76.11	87.52	87.09	87.13
	32	74.14	74.37	75.21	85.18	85.54	84.53
	40	73.80	73.94	73.14	82.22	83.81	82.36

ECG	16	71.44	71.15	69.63	80.40	79.74	80.23
	24	74.10	74.41	73.30	82.29	82.13	81.53
	32	70.89	72.56	72.18	82.26	81.76	80.28
	40	68.37	67.51	66.30	81.87	80.54	81.47

EEG + ECG	16	78.29	78.35	78.58	**89.74**	89.04	**89.28**
	24	81.75	80.64	79.44	88.51	**90.13**	89.94
	32	78.67	77.18	78.43	88.47	88.03	87.27
	40	75.13	75.72	74.54	88.36	86.83	87.70

Bold values are the best values obtained.

**Table 4:** The calculation of accuracy, AUC, and RR in various scenarios of wavelet descriptor and subset feature selection. The results of SVM and improved SVM classifiers are compared to detect arousal events.

Signal type	No. of features	SVM			Optimized SVM		
		ACC (%)	AUC (%)	RR (%)	ACC (%)	AUC (%)	RR (%)
EEG	4	74.62	74.70	73.91	81.32	79.56	78.38
	8	79.73	78.04	78.81	82.81	83.60	82.32
	12	75.08	76.50	76.82	83.24	82.36	81.52
	16	75.70	75.11	74.89	81.38	81.28	80.24

ECG	4	72.22	72.09	72.49	80.38	79.86	79.24
	8	77.29	77.83	76.43	81.82	82.36	81.62
	12	73.21	73.37	74.97	81.90	81.44	80.68
	16	72.04	73.37	73.84	80.63	80.20	80.11

EEG + ECG	4	78.13	78.04	78.77	86.46	87.66	86.80
	8	81.65	80.22	79.05	**89.73**	**90.34**	**89.76**
	12	75.61	76.31	76.49	88.47	88.08	86.42
	16	75.81	75.05	74.25	86.67	86.33	85.18

Bold values are the best values obtained.

**Table 5:** The Lyapunov exponent descriptor was used to calculate accuracy, AUC, and RR in various scenarios of the subset feature selection. The results of SVM and improved SVM classifiers are compared to detect arousal events.

Signal type	No. of features	SVM			Optimized SVM		
		ACC (%)	AUC (%)	RR (%)	ACC (%)	AUC (%)	RR (%)
EEG	8	73.03	73.60	71.54	80.47	80.99	80.76
	12	78.51	79.10	79.83	83.35	84.71	83.02
	16	74.98	75.75	75.92	84.27	83.54	81.43
	20	76.15	74.33	76.78	82.72	82.07	81.11

ECG	8	71.34	71.86	71.04	80.19	80.14	80.24
	12	76.23	76.07	76.66	82.74	83.05	82.62
	16	73.45	73.65	73.69	81.73	81.57	80.68
	20	72.14	72.62	72.87	80.78	79.66	79.11

EEG + ECG	8	76.54	76.46	76.75	90.14	89.23	90.80
	12	79.06	78.81	79.71	91.42	**91.70**	**91.56**
	16	77.80	77.67	77.12	**91.87**	91.46	90.48
	20	73.11	73.24	73.49	90.93	90.83	89.31

Bold values are the best values obtained.

**Table 6:** The performance of models for arousal detection and sleep stage classification using metrics such as accuracy and computational complexity.

Author	Procedure type	Signal (s)	Accuracy (%)	Computational complexity
Supratak et al. [56]	Sleep stage scoring	Raw single-channel EEG	85.50	High
Willemen et al. [57]	Sleep stage classification	Cardiorespiratory and movement	86.75	Moderate
Fonseca et al. [58]	Sleep stage detection	Cardiorespiratory	87.38	High
Alickovic et al. [59]	Sleep stage classification	EEG	91.10	Moderate
Fernández-Varela et al. [60]	Arousals monitoring	Polysomnographic	86.00	Low
Li and Guan [25]	Arousals monitoring	Polysomnographic	92.80	High
Mousavi et al. [26]	Sleep stage detection	Single-channel EEG signals	93.55	High
Our proposed approach	Sleep stage recognition	EEG and ECG	93.76	Low
	Arousals detection	EEG and ECG	93.23	Low

## Data Availability

All the codes are available from the corresponding author upon request. The data that support the findings of this study were derived from the resources available in the public domain (https://sleepdata.org/datasets/shhs) and (https://archive.physionet.org/physiobank/database/challenge/2018).

## References

[1] Christensen J., Noel M., Mychasiuk R. (2019). Neurobiological mechanisms underlying the sleep-pain relationship in adolescence: a review. *Neuroscience & Bio behavioral Reviews*.

[2] Yang X., Shah S. A., Ren A. (2018). Monitoring of patient s suffering from REM sleep behavior disorder. *IEEE Journal of Electromagnetics, RF and Microwaves in Medicine and Biology*.

[3] Bhattacharjee A., Sa ha S., Fattah S. A., Zhu W. P., Ahmad M. O. (2019). Sleep apnea detection based on rician modeling of feature variation in multiband eeg signal. *IEEE journal of biomed ical and health informatics*.

[4] Jang S. H., Kim S. H., Kwon Y. H. (2018). Excessive daytime sleepiness and injury of the ascending reticular activating system following whiplash injury. *Frontiers in Neuroscience*.

[5] Ma Y., Sun S., Zhang M. (2020). Electrocardiogram-based sleep analysis for sleep apnea screening and diagnosis. *Sleep and Breathing*.

[6] Kongwudhikunakorn S., Kiatthaveephong S., Thanontip K. (2021). A pilot study on visually stimulated cognitive tasks for EEG-based dementia recognition. *IEEE Transactions on Instrumentation and Measurement*.

[7] van Boxtel G. J., Cluitmans P. J., Raymann R. J. (2018). Heart rate variability, sleep, and the early detection o f post-traumatic stress disorder. *Sleep and Combat-Related post Traumatic Stress Disorder*.

[8] Cooray N., Andreotti F., Lo C., Symmonds M., Hu M. T., De Vos M. (2019). Detection of REM sleep behaviour disorder by automated polysomnography analysis. *Clinical Neurophysiology*.

[9] Schenck C. H., Boeve B. F., Mahowald M. W. (2013). Delayed emergence of a parkinsonian disorder or dementia in 81% of older men initially diagnosed with idiopathic rapid eye movement sleep behavior disorder: a 16-year update on a previously reported series. *Sleep Medicine*.

[10] Ding X., Yan B. P., Karlen W., Zhang Y. T., Tsang H. K. (2020). Pulse transit time based respiratory rate estimation with singular spectrum analysis. *Medical, & Biological Engineering & Computing*.

[11] Whitehurst L. N., Chen P. C., Naji M., Mednick S. C. (2020). New directions in sleep and memory research: the role of autonomic activity. *Current Opinion in Behavioral Sciences*.

[12] Fernandez L. M. J., Lüthi A. (2020). Sleep spindles: mechanisms and functions. *Physiological Reviews*.

[13] Lofaso F., Goldenberg F., d’Ortho M. P., Coste A., Harf A. (1998). Arterial blood pressure response to transient arousals from NREM sleep in nonapneic snorers with sleep fragmentation. *Chest*.

[14] Walsh J. H., Visser C., Maddison K., Bharat C., Hillman D. R., Eastwood P. R. (2019). The effect of temazepam on assessment of severity of obstructive sleep apnea by polysomnography. *Sleep and Breathing*.

[15] Halász P., Terzano M., Parrino L., Bódizs R. (2004). The nature of arousal in sleep. *Journal of Sleep Research*.

[16] Lachapelle P., Cascon J., Pamidi S., Kimoff R. J. (2019). Accuracy of portable devices in sleep apnea using oximetry-derived heart rate increases as a surrogate arousal marker. *Sleep and Breathing*.

[17] Fonod R. (2022). DeepSleep 2.0: automated sleep arousal segmentation via deep learning. *A&I*.

[18] Basner M., Griefahn B., Müller U., Plath G., Samel A. (2007). An ECG-based algorithm for the automatic identification of autonomic activations associated with cortical arousal. *Sleep*.

[19] Xu X., Ni L., Wang R. (2016). A neural network model of spontaneous up and down transitions. *Nonlinear Dynamics*.

[20] Olsen M., Schneider L. D., Cheung J. (2018). Automatic, electrocardiographic-based detection of autonomic arousals and their association with cortical arousals, leg movements, and respiratory events in sleep. *Sleep*.

[21] Pillar G., Bar A., Betito M. (2003). An automatic ambulatory device for detection of AASM defined arousals from sleep: the WP100. *Sleep Medicine*.

[22] Warrick P., Nabhan Homsi M. Sleep arousal detection from polysomnography using the scattering transform and recurrent neural networks.

[23] Sors A., Bonnet S., Mirek S., Vercueil L., Payen J. F. (2018). A convolutional neural network for sleep stage scoring from raw single-channel EEG. *Biomedical Signal Processing and Control*.

[24] Vilamala A., Madsen K. H., Hansen L. K. Deep convolutional neural networks for interpretable analysis of EEG sleep stage scoring.

[25] Li H., Guan Y. (2021). DeepSleep convolutional neural network allows accurate and fast detection of sleep arousal. *Communications biology*.

[26] Mousavi Z., Yousefi Rezaii T., Sheykhivand S., Farzamnia A., Razavi S. N. (2019). Deep convolutional neural network for classification of sleep stages from single-channel EEG signals. *Journal of Neuroscience Methods*.

[27] Li H., Cao Q., Zhong Y., Pan Y. Sleep arousal detection using end-to-end deep learning method based on multi-physiological signals.

[28] Tsinalis O., Matthews P. M., Guo Y. (2016). Automatic sleep stage scoring using time-frequency analysis and stacked sparse autoencoders. *Annals of Biomedical Engineering*.

[29] Macias E., Morell A., Serrano J., Vicario J. L. Knowledge extraction based on wavelets and DNN for classification of physiological signals: arousals case.

[30] Zhou G., Li R., Zhang S., Wang J., Ma J. (2020). Multimodal sleep signals-based automated sleep arousal detection. *IEEE Access*.

[31] Ugur T. K., Erdamar A. (2019). An efficient automatic arousals detection algorithm in single channel EEG. *Computer Methods and Programs in Biomedicine*.

[32] Karimi J., Asl B. M. (2021). Automatic detection of non-apneic sleep arousal regions from polysomnographic recordings. *Biomedical Signal Processing and Control*.

[33] Hekmatmanesh A., Mohammadi Asl R., Wu H., Handroos H. (2019). EEG control of a bionic hand with imagination based on chaotic approximation of largest Lyapunov exponent: a single trial BCI application study. *IEEE Access*.

[34] Nankani D., Parabattina B., Baruah R. D., Das P. K. R-peak detection from ECG signals using fractal based mathematical morphological operators.

[35] Reyes-Sanchez E., Alba A., Mendez M. O., Milioli G., Parrino L. (2016). Spectral entropy analysis of the respiratory signal and its relationship with the cyclic alternating pattern during sleep. *International Journal of Modern Physics C*.

[36] Sharma M., Bhurane A. A., Acharya U. R. (2022). An expert system for automated classification of phases in cyclic alternating patterns of sleep using optimal wavelet-based entropy features. *Expert Systems*.

[37] Halász P. (2016). The K-complex as a special reactive sleep slow wave–a theoretical update. *Sleep Medicine Reviews*.

[38] Dahal P., Avagyan M., Skardal P. S., Blaise H. J., Ning T. Characterizing chaotic behavior of REM sleep EEG using lyapunov exponent.

[39] Al-Salman W., Li Y., Wen P., Diykh M. (2018). An efficient approach for EEG sleep spindles detection based on fractal dimension coupled with time frequency image. *Biomedical Signal Processing and Control*.

[40] Kantar T., Erdamar A. Continuous wavelet transform based method for detection of arousal.

[41] Liang Y., Leung C., Miao C., Wu Q., McKeown M. J. Automatic sleep arousal detection based on c-elm.

[42] Chien Y. R., Wu C. H., Tsao H. W. (2021). Automatic sleep-arousal detection with single-lead EEG using stacking ensemble learning. *Sensors*.

[43] Banluesombatkul N., Ouppaphan P., Leelaarporn P. (2021). MetaSleepLearner: a pilot study on fast adaptation of bio-signals-based sleep stage classifier to new individual subject using meta-learning. *IEEE Journal of Biomedical and Health Informatics*.

[44] de Melo V. V., Banzhaf W. (2018). Drone Squadron Optimization: a novel self-adaptive algorithm for global numerical optimization. *Neural Computing & Applications*.

[45] Cortes C., Vapnik V. (1995). Support-vector networks. *Machine Learning*.

[46] Keelawat P., Thammasan N., Numao M., Kijsirikul B. (2021 Jan). A comparative study of window size and channel arrangement on EEG-emotion recognition using deep CNN. *Sensors*.

[47] Yakovleva T. V., Kutepov I. E., Karas A. Y. (2020). EEG analysis in structural focal epilepsy using the methods of nonlinear dynamics (Lyapunov exponents, Lempel–Ziv complexity, and multiscale entropy). *The Scientific World Journal*.

[48] Aspuru J., Ochoa-Brust A., Félix R. A. (2019). Segmentation of the ECG Signal by means of a linear regression algorithm. *Sensors*.

[49] Horner R. L. (2016). Physiological effects of sleep on the cardiovascular system. *Sleep Apnea*.

[50] Noda A., Yasuma F., Miyata S., Iwamoto K., Yasuda Y., Ozaki N. (2019). Sleep fragmentation and risk of automobile accidents in patients with obstructive sleep apnea—sleep fragmentation and automobile accidents in OSA. *Health*.

[51] Jaimchariyatam N., Rodriguez C. L., Budur K. (2015). Sleep-related cortical arousals in adult subjects with negative polysomnography. *Sleep and Breathing*.

[52] Kim J. B., Seo B. S., Kim J. H. (2019). Effect of arousal on sympathetic overactivity in patients with obstructive sleep apnea. *Sleep Medicine*.

[53] Amatoury J., Azarbarzin A., Younes M., Jordan A. S., Wellman A., Eckert D. J. (2016). Arousal intensity is a distinct pathophysiological trait in obstructive sleep apnea. *Sleep*.

[54] Subramanian S., Chakravarty S., Chamadia S. Arousal detection in obstructive sleep apnea using physiology-driven features.

[55] Berry R. B., Brooks R., Gamaldo C. E. (2015). Should the arousal scoring rule be changed?. *Journal of Clinical Sleep Medicine*.

[56] Supratak A., Dong H., Wu C., Guo Y. (2017). DeepSleepNet: a model for automatic sleep stage scoring based on raw single-channel EEG. *IEEE Transactions on Neural Systems and Rehabilitation Engineering*.

[57] Willemen T., Van Deun D., Verhaert V. (2014). An evaluation of cardiorespiratory and movement features with respect to sleep-stage classification. *IEEE journal of biomedical and health informatics*.

[58] Fonseca P., den Teuling N., Long X., Aarts R. M. (2017). Cardiorespiratory sleep stage detection using conditional random fields. *IEEE journal of biomedical and health informatics*.

[59] Alickovic E., Subasi A. (2018). Ensemble SVM method for automatic sleep stage classification. *IEEE Transactions on Instrumentation and Measurement*.

[60] Fernández-Varela I., Alvarez-Estevez D., Hernández-Pereira E., Moret-Bonillo V. (2017). A simple and robust method for the automatic scoring of EEG arousals in polysomnographic recordings. *Computers in Biology and Medicine*.

[61] Duan L., Li M., Wang C. (2021). A novel sleep staging network based on data adaptation and multimodal fusion. *Frontiers in Human Neuroscience*.

[62] Touil M., Bahatti L., El Magri A. (2022). Sleep’s depth detection using electroencephalogram signal processing and neural network classification. *Journal of Medical Artificial Intelligence*.

[63] Chien Y. R. (2018). Wavelet packet transform-based anti-jamming scheme with new threshold selection algorithm for GPS receivers. *Journal of the Chinese Institute of Engineers*.

[64] Rezaee K., Khosravi M. R., Jabari M., Hesari S., Anari M. S., Aghaei F. (2022). Graph convolutional network-based deep feature learning for cardiovascular disease recognition from heart sound signals. *International Journal of Intelligent Systems*.

[65] Rezaee K., Khosravi M. R., Moghimi M. K. (2022). Intelligent elderly people fall detection based on modified deep learning deep transfer learning and IoT using thermal imaging-assisted pervasive surveillance. *Intelligent Healthcare*.

